# A Web-Based Program About Sustainable Development Goals Focusing on Digital Learning, Digital Health Literacy, and Nutrition for Professional Development in Ethiopia and Rwanda: Development of a Pedagogical Method

**DOI:** 10.2196/36585

**Published:** 2022-12-05

**Authors:** Katarina Bälter, Feben Javan Abraham, Chantal Mutimukwe, Reuben Mugisha, Christine Persson Osowski, Olle Bälter

**Affiliations:** 1 Department of Public Health School of Health, Care and Social Welfare Mälardalen University Västerås Sweden; 2 Department of Medical Epidemiology and Biostatistics Karolinska Institutet Stockholm Sweden; 3 Department of Media Technology and Interaction Design Kungliga Tekniska Högskolan Royal Institute of Technology Stockholm Sweden

**Keywords:** digital learning, digital health literacy, sustainable development goals, public health, nutrition, question-based learning, open learning initiative, Rwanda, Ethiopia

## Abstract

**Background:**

East African countries face significant societal challenges related to sustainable development goals but have limited resources to address these problems, including a shortage of nutrition experts and health care workers, limited access to physical and digital infrastructure, and a shortage of advanced educational programs and continuing professional development.

**Objective:**

This study aimed to develop a web-based program for sustainable development with a focus on digital learning, digital health literacy, and child nutrition, targeting government officials and decision-makers at nongovernmental organizations (NGOs) in Ethiopia and Rwanda.

**Methods:**

A web-based program—OneLearns (Online Education for Leaders in Nutrition and Sustainability)—uses a question-based learning methodology. This is a research-based pedagogical method developed within the open learning initiative at Carnegie Mellon University, United States. Participants were recruited during the fall of 2020 from ministries of health, education, and agriculture and NGOs that have public health, nutrition, and education in their missions. The program was conducted during the spring of 2021.

**Results:**

Of the 70 applicants, 25 (36%) were selected and remained active throughout the entire program and filled out a pre- and postassessment questionnaire. After the program, of the 25 applicants, 20 (80%, 95% CI 64%-96%) participants reported that their capacity to drive change related to the sustainable development goals as well as child nutrition in their organizations had increased *to large extent* or *to a very large extent*. Furthermore, 17 (68%, 95% CI 50%-86%) and 18 (72%, 95% CI 54%-90%) participants reported that their capacity to drive change related to digital health literacy and digital learning had increased *to a large extent* and *to a very large extent*, respectively.

**Conclusions:**

Digital learning based on a question-based learning methodology was perceived as a useful method for increasing the capacity to drive change regarding sustainable development among government officials and decision-makers at NGOs in Ethiopia and Rwanda.

## Introduction

### Background

East African countries face significant societal challenges covered by the global sustainable development goals (SDG) [1]. A total of 7 million people are at risk of starvation and >33 million people face acute food insecurity [1]. Furthermore, the pooled prevalence of chronic undernutrition among children aged <five years was 33% (95% CI 33-36%) in East Africa, ranging from 22% in Kenya to 53% in Burundi [2]. At the same time, East African countries have limited resources to address these problems [3], including a shortage of nutrition experts and health care workers [4,5], limited access to infrastructure, and a shortage of advanced educational programs and continuing professional development [4]. The SDGs were introduced to “achieve a better and sustainable future for all” [6]. To achieve the SDGs in general, good health and well-being (goal 3) and quality education (goal 4) in particular, effective education initiatives are needed [7]. Digital learning has the potential to overcome the lack of professional education and development [8], especially during the COVID-19 pandemic.

Although information and communication technology (ICT) is central to meeting new skills and training demands in most low- and middle-income countries [9], internet connectivity in most African institutions, especially those in rural and semiurban areas, is limited, expensive, unstable, or poorly managed [10,11]. Even though these countries are lagging in the adoption and implementation of effective digital learning tools [8], the advantages of digital learning in achieving the SDGs in East Africa are clear. In this context, digital learning, or e-learning, is based on the use of electronic media and devices as tools to improve access to training, communication, and interaction as well as to facilitate the adoption of new ways of understanding and learning [12]. However, research on digital learning issues in East Africa is scarce [4]. Pedagogical research from high-income countries may potentially be inapplicable to a low-resource context because of differences in teachers’ educational backgrounds and experiences. In addition, local cultural contexts and perceptions must be considered [13].

Similar to digital learning, digital health literacy is a fundamental aspect of any country’s economic development and a major contributor to the realization of SDGs [14]. Digital literacy is the ability to understand and use information in multiple formats from a wide variety of sources when it is presented via electronic sources [15]. Digital health literacy is the ability to seek, understand, and appraise health information from electronic sources and apply the knowledge gained to address or solve health problems [16]. However, most individuals holding decision-making positions in East Africa lack basic knowledge of ICT and related training [17]. Training in information technology literacy is therefore essential for the successful integration of digital health initiatives into existing health care services in low- and middle-income countries [18].

Rwanda encourages web-based learning and the digitization of several other service sectors, such as health care, commerce, and governance [19]. However, academics and students have been reluctant to fully engage in web-based education, mainly because most web-based platforms and applications fail to reach the core of the learning crisis in Rwanda, because some learning materials are difficult to comprehend, and there is no documented evidence of their effectiveness compared with physical classrooms. In Ethiopia, digitalization of education in schools and universities is still in its infancy. This is primarily because of the lack of infrastructure development in ICT and insufficient human resources with knowledge and training, creating barriers to move forward [20]. Therefore, we developed the OneLearns (Online Education for Leaders in Nutrition and Sustainability) program aimed at increasing human resource capacity within the digitalization of the health and education fields. Specifically, OneLearns capitalizes on the documented evidence of question-based learning methodology as an effective and time-saving method for both students and teachers [21].

### Objectives

The OneLearns program targeted government officials and decision-makers in nongovernmental organizations (NGOs) in Ethiopia and Rwanda and covered the SDGs with a focus on digital learning, digital health literacy, and nutrition. We described the development of the OneLearns program, learning objectives, question-based learning methodology, process of recruiting participants, and the results of the program evaluation. Finally, we highlighted some lessons learned and ways to improve the program in the future.

## Methods

### The Teaching Team

The OneLearns program was designed and developed by a team of 6 experts at the KTH Royal Institute of Technology and Mälardalen University, Sweden, between September 2020 and December 2020. The team included a professor, 2 associate professors, a postdoctoral researcher, a PhD student, and a research assistant with a master’s degree. The team had expertise in different domains including digital learning technologies, digital health literacy, sustainable development, public health, and nutrition. The program was based on the team’s research fields, but to ensure that the program would be relevant for the target participants from Rwanda and Ethiopia, the content was discussed with research colleagues from Rwanda and Ethiopia, including members of the teaching team from Ethiopia and Rwanda.

### Question-Based Learning Methodology

**Figure 1:**
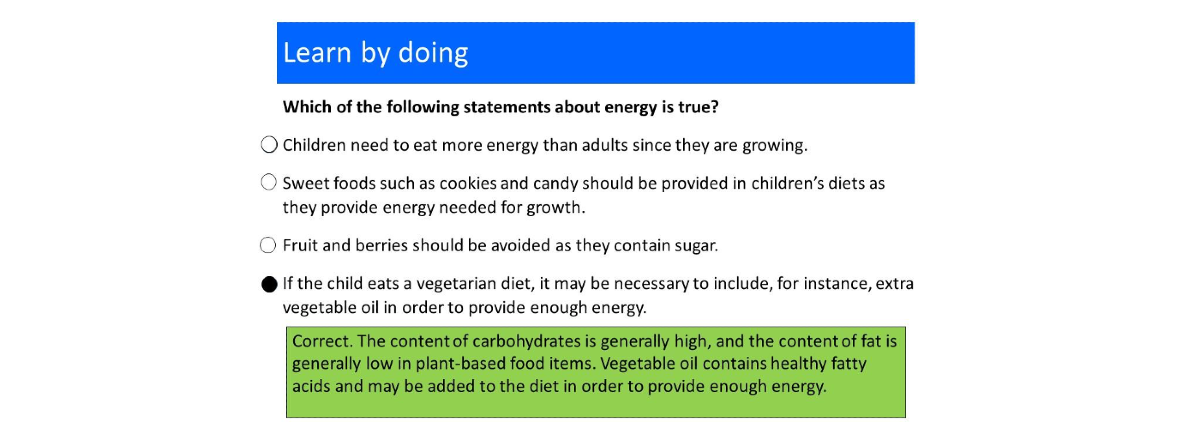
An example of a formative question from the module on Child nutrition in the web-based material. The green rectangle shows feedback that the participant received after selecting the correct answer. It serves the purpose of reinforcing the correct answer and teaches the student something new about the topic.

**Figure 2:**
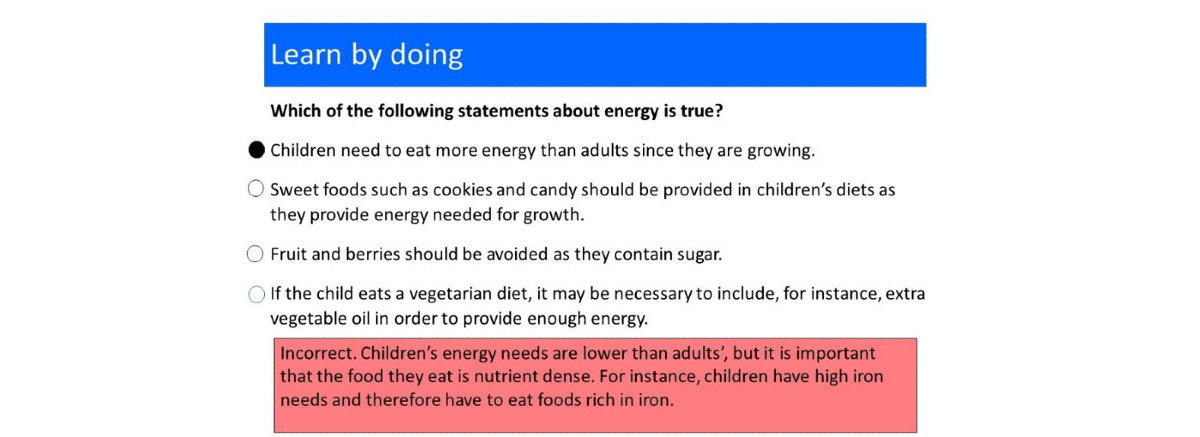
An example of a formative question from the module on Child nutrition in the web-based material. The red rectangle shows feedback that the participant received after selecting an incorrect answer and targets common misconceptions associated with the wrong answer, thus contributing to the learning process.

The program used a question-based learning methodology in which the learning material was organized around formative questions linked to one or more skills, which in turn were linked to the learning objectives [21]. More concrete examples from the program are shown in Figures 1 and 2, but in general, this question-based learning methodology is a research-based pedagogical method developed within the open learning initiative at Carnegie Mellon University, United States [22]. The foundation of the methodology is that questions with constructive feedback are scattered over the learning material to stimulate students to engage with it. The methodology is used both in fully web-based courses and on campus, often in a flipped classroom setting [23], meaning that students worked with the web-based question-based material before coming to class, and the teacher used the learning data accumulated from the students’ activities in the learning material to plan the lecture. This type of active learning is 6 times more efficient than reading and watching videos [24]. The methodology used basic Bayesian hierarchical models to predict student mastery [25], and when used repeatedly and refined with the data from each course iteration, this method became very effective for learners. In a randomized case-control study on campus at Carnegie Mellon University, Pittsburgh, United States with this type of web-based learning material, learning time was reduced by 50%, while maintaining the learning outcomes compared with a parallel traditional course [22]. For newly developed material (without iteratively improved material, such as in this project), the reduction in learning time was estimated to be 25%, with maintained learning outcomes [21]. This method was therefore considered suitable for the target participants, as they are professionals with full-time work and, hence, do not have much time.

### Program Design and Development

The program was designed for participants’ self-paced studies of digital material followed by web-based video seminars at the end of each unit, in line with the principle of the flipped classroom. The program comprised 3 units (Multimedia Appendix 1). The first unit was titled “Web-based learning” and subdivided into 2 modules: “Becoming a web-based learner” and “Effective digital learning.” The second unit focused on “Digital health literacy” and included the 2 modules “Introduction to digital health literacy” and “Fundamentals of digital literacy in contemporary health care.” Finally, the third unit, “Nutrition and the SDGs” consisted of a module “Nutrition and sustainability.”

To ensure that the material was relevant to the target group and countries, half (3/6, 50%) of the OneLearns teachers were from Ethiopia or Rwanda. Moreover, policy documents and other teaching materials were reviewed to ensure that the topics included in the program were relevant for and adjusted to their corresponding countries. The first step in the development of the program was to determine the learning objectives and related skills for each module. In total, 17 learning objectives and 47 skills were identified; an example of a skill can be as follows: “Explain the pros and cons of web-based learning.” Thereafter, program designers formulated a series of formative questions for each skill. The formative questions formed the backbone of the digital learning material [22] and were designed as multiple-choice questions, ordering questions, select-all-that-apply questions, and drag-and-drop questions. The idea behind the formative questions was to engage students in collaborative tasks that supported authentic practice with the concepts and skills they were learning. The questions aimed for, at minimum, the *understanding* level of the Bloom taxonomy of learning, which is a hierarchical model that categorizes learning objectives into varying levels of complexity, and the level of “understanding” is the second lowest level [26]. Each formative question generated automatic feedback to the participant regarding whether the answer was correct or incorrect. Feedback reinforced the correct answer and targeted common misconceptions associated with incorrect answers (Figures 1 and 2). Construction of the formative questions with relevant answering alternatives and constructive feedback was based on the principles developed by Glassey and Bälter [27] (Textbox 1).

Next, the program designers compiled the digital learning material in the form of short texts, images, simulations, short videos, and working examples to support the learning process, ensuring that the participants could answer the formative questions. Part of the learning material was reused from open sources (websites, video clips, and so on, especially from Athabasca University, Stanford University, and Carnegie Mellon University), and the rest was based on the teaching team’s own research. Finally, questions for the final assessment tests were formulated. The final assessment tests took place at the end of modules B, C, D, and E (Multimedia Appendix 1) and were referred to as module tests. No feedback besides right or wrong answers was offered, but the participants had the opportunity to retake each module test up to three times for revision and refinement. The final score was either pass or fail, and 100% of correct answers were required to pass the tests in modules B, C, and D, whereas it was 80% for module E because that module had more questions than the others. The module tests were either autocorrected or graded manually, as in the case of essay or short-answer questions.

**The construction of formative questions with relevant answering alternatives and constructive feedback was based on the principles developed by Glassey and Bälter [27].**

**Principles of good questions**

- Question is from the program domain (ie, related to the learning objectives of the program).
- Question is targeted toward a misconception.
- Question is not based on a reference lookup.
- Question is reasonable to solve without external systems.

**Principles of good answering alternatives**

- Three or more answer alternatives are provided.
- Answer alternatives are plausible and linked to the misconception.
- Answer alternatives are formulated to maximize readability.

**Principles of good feedback**

- Feedback is constructive.
- Feedback is unique and provided for each answer alternative.
- Feedback for answer alternatives does not reveal the answer.

### Recruitment Process

The recruitment process continued between September 2020 and December 2020, targeting potential participants from various ministries with a focus on the ministries of health, education, and agriculture and NGOs with public health, nutrition, and education in their missions. We collaborated with the embassies of Rwanda and Ethiopia in Stockholm, who distributed calls for applications to ministries and NGOs in their respective countries. To reach the different ministries, the information had to go via the ministry of foreign affairs, whereas the NGOs were reached via less formal networks. In addition, we sent information to our own network of NGO contact persons and to the Swedish embassies in Ethiopia and Rwanda, which they then shared further.

We received 70 applications, including 42 from Ethiopia and 28 from Rwanda. Applicants were screened and scored according to the professional field, leadership experience, educational background, current job position, and their potential for decision-making, as well as the ability to express oneself in English. Furthermore, we aimed to achieve a gender balance of close to 1:1 ratio for men and women. On the basis of applicant scoring, 27 applicants were selected for the second phase of recruitment, where web-based interviews were conducted primarily to verify sufficient communication and verbal skills in English. Although 3 (11%) participants were recognized as having less experience communicating orally in English, none of them were excluded. Of the 27 participants, 13 (48%) were men, 14 (52%) women, 15 (56%) from Ethiopia, and 12 (44%) from Rwanda. Of these, 26 (96%) successfully registered to the program, but 1 (4%) requested withdrawal from the program after the registration, leaving 25 active participants. For the 2 (7%) participants who did not start the program, one reported a change in work circumstances that conflicted with the program’s schedule, while the other did not communicate a reason and failed to register completely.

### Program Delivery and Timeline

**Figure 3:**
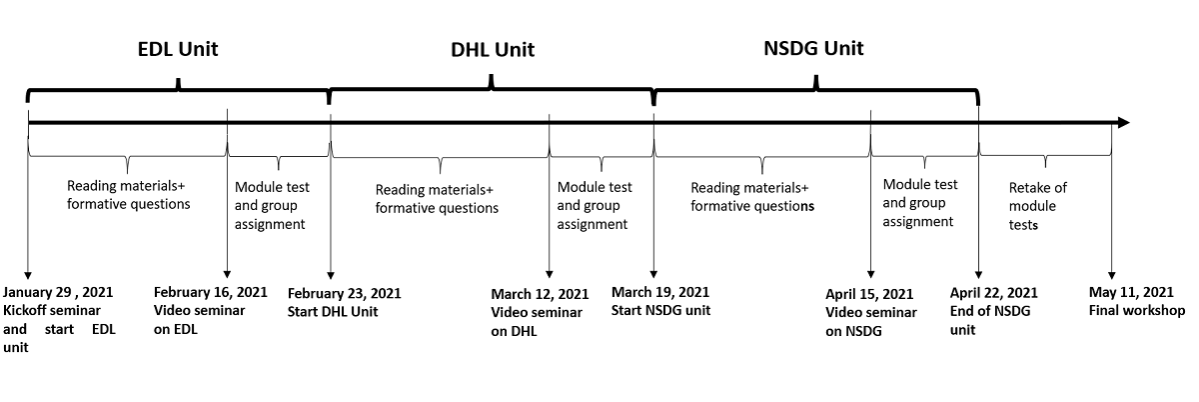
Timeline for the web-based program OneLearns (Online Education for Leaders in Nutrition and Sustainability). The program ran between January 29, 2021, and May 11, 2021, and comprised 3 modules. DHL: digital health literacy; EDL: Effective Digital Learning; NSDG: nutrition and the sustainable development goal.

The program continued between January 29, 2021, and May 11, 2021 (Figure 3), and it was conducted entirely on the web owing to the ongoing COVID-19 pandemic. The original plan was to invite a representative subgroup of participants to Sweden at the beginning of the program and conduct the final workshop on-site in Ethiopia and Rwanda, but this part of the program was canceled as the pandemic did not allow travel. Each participant was expected to spend approximately 40 hours of study time during the program, and the program design allowed a self-paced learning approach to a large extent.

Each unit was supposed to be completed within approximately 25 days. The participants began by working with the digital material and answering formative questions, followed by a web-based video seminar with teachers from Sweden and a final group assignment and module test. In the absence of physical interaction, it was important to ensure that the participants interacted and networked with each other. Therefore, 1 group assignment per program unit was included. Reminders were sent weekly with deadlines, including a general group progress report. The program ended on May 11, 2021, with a full-day web-based workshop and included invited speakers from Sweden, Rwanda, and Ethiopia and “learning by doing” activities. Unlimited data packages were provided to all participants in Ethiopia and Rwanda to ensure that they could be involved in the workshop. In addition, the participants in Rwanda were invited to meet in a large conference room at a hotel in Kigali and take part in the workshop from there, whereas the COVID-19 pandemic restrictions in Ethiopia did not allow for physical gatherings at that point in time.

### Means of Digital Communications and Interaction

The predominant means of communicating program information, progress updates, and deadline reminders to the participants was email. For less formal communication, that is, sharing scientific articles or pertinent seminars, a WhatsApp group was set up so that the participants could network as well. Google Docs was used for group assignment submissions, and teachers used the comment function to provide feedback on assignments. The initial kickoff, video seminars at the end of each module, and final workshop were conducted via Zoom (Zoom Video Communications, Inc). Moreover, during the final workshop, a Miro board, a web-based creative collaboration platform [28], was used for brainstorming, and the sticky note function was used to share ideas on a web-based and collaborative board (Multimedia Appendix 2).

### Data Collection and Analysis

Pre- and postassessment surveys were administered to assess the participants’ knowledge, capacity, and experience both *before* and *after* the program. These web-based surveys included questions provided by the grant provider and the national foreign aid agency (Swedish Institute). To the best of our knowledge, these questions have not been validated but are used in all programs that they support. The surveys were distributed using the survey software Survey Generator and included automatic reminders to nonresponders. The first survey had 17 questions, whereas the latter had 9 (Multimedia Appendices 3 and 4).

**Figure 4:**
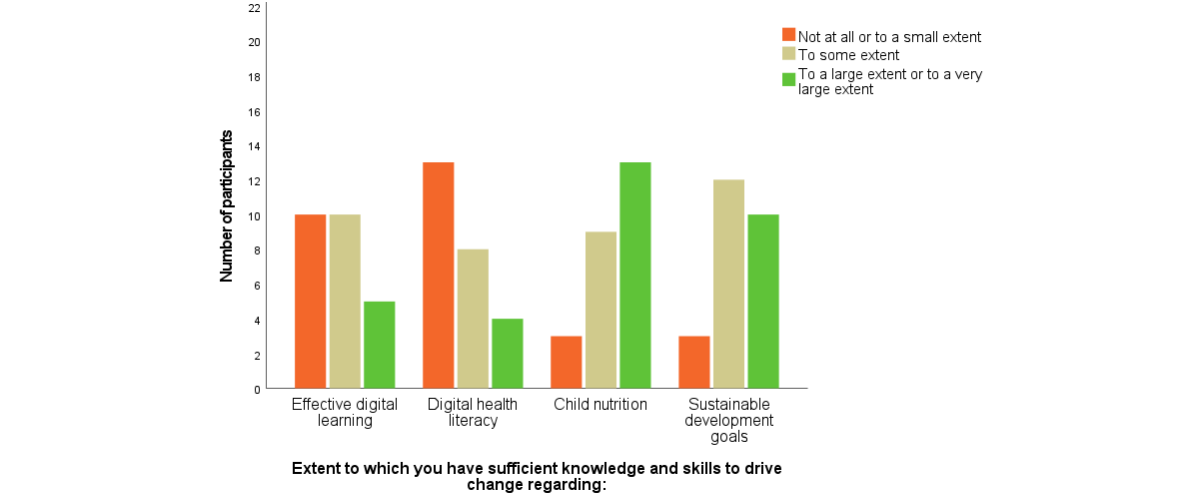
Participants self-reported knowledge and skills to drive change before the program for various topics.

**Figure 5:**
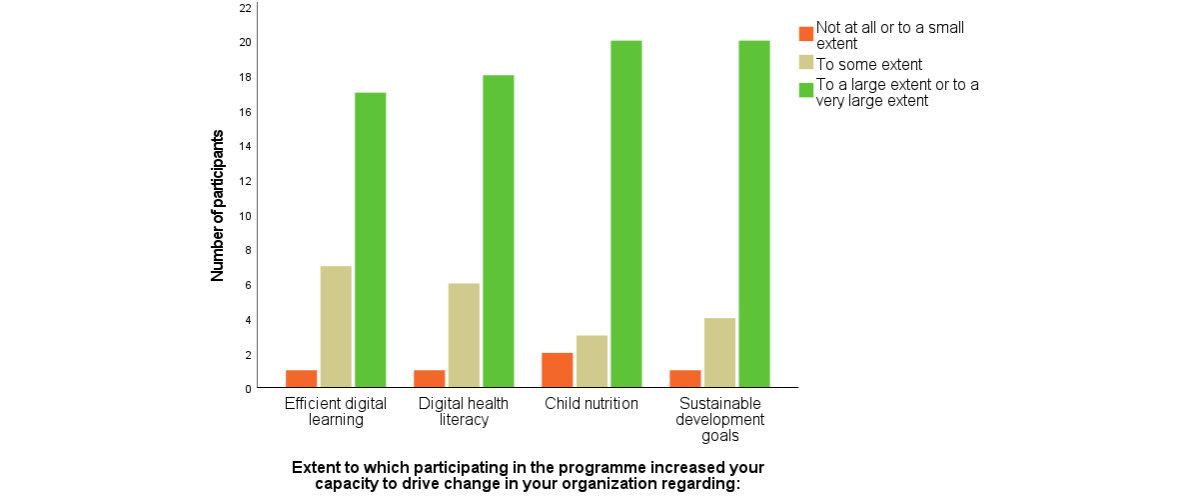
Participants self-reported capacity to drive change after the program for various topics.

The overall aim of the preassessment survey was to capture the participants’ demographic and professional profiles and their own perception of their capacity in the various topics, that is, the current level of knowledge and confidence to bring about change. Questions such as “To what extent do you have sufficient knowledge and skills to drive change regarding digital learning” were used, with a nominal scale with options “not at all,” “to a small extent,” “to some extent,” “to a large extent,” or “to a very large extent” or “not applicable.” Owing to the small number of participants, categories were merged into three main categories (Figures 4 and 5), (1) *not at all and to a small extent*, (2) *to some extent*, and (3) *to a large or to a very large extent*. Descriptive data were generated by Survey Generator from Alstra or analyzed using IBM SPSS 26 Statistics or Microsoft Excel Analysis ToolPak.

To evaluate the program material, an end-of-unit evaluation with open-ended questions was sent to the 25 participants after the *Efficient digital learning* and the *Nutrition and sustainability* units were completed, to which 12 (48%) and 18 (72%) participants responded, respectively. Questions such as “Which section did you learn the most from?’ and ‘What did you think was missing from the unit?” were used to identify areas of improvement for future programs; however, an analysis of the results was beyond the scope of this study.

Data on the performance of the participants, that is, their engagement with the learning material and learning objectives, were automatically collected by the open learning initiative system. Response rates and answering patterns of the formative questions were assessed; for example, how many times different answer alternatives were selected for the multiple-choice questions. In addition, based on the data that the open learning initiative system collected from participant engagement with the program material, it automatically generated learning curves for each of the 47 skills in the program.

### Ethical Considerations

Paragraph 2 of the Swedish Ethical Review Authority states that ethics approval is only necessary if a project collects sensitive personal data from the participants, involves physical procedures, uses a method that will impact the participants physically or physiologically or if there is a risk of harm, or involves biological material [29]. None of this was the case in the OneLearns program; therefore, we did not apply for ethics approval. Participation in the web-based program was voluntary, and it was possible to discontinue the program at any point without informing why. All participants received written and oral information about the program before it started, and filling out the pre- and postassessment questionnaires was considered as consent to help evaluate and improve the educational program.

## Results

### The Participants and Program Completion Rate

Table 1 shows demographic characteristics of the 25 participants enrolled in the program. Most participants (22/25, 88%) were aged <40 years, the gender distribution was almost equal with 13 (52%) women and 12 (48%) men, and 13 (52%) were from Ethiopia and 12 (48%) from Rwanda. Almost half (12/25, 48%) of the participants were working in governmental institutions, followed by 10 (40%) working in NGOs, and finally 3 (12%) in private organizations and other organizations that engaged with civil society; for example, project management or consultancy or other sectors. Most of the participants had educational backgrounds related to health, such as public health (8/25, 32%), nutrition (5/25, 20%), and the medical field (3/25, 12%). The remaining participants had backgrounds in agriculture (2/25, 8%) or other various backgrounds (7/25, 28%), including software, IT, project management and business administration.

All 25 participants remained active and participated throughout the entire program, but 3 (12%) did not complete all the tasks required by the program, such as formative questions and module tests. Divided by units, of the 25 participants, 24 (96%) successfully completed all the requirements of units 1 and 2, and 23 (92%) completed those of unit 3. Participants who completed all the tasks were given a certificate of completion, and the 3 (%) participants who remained active but did not complete all the tasks required by the program received a certificate of participation.

**Table 1:** Characteristics of the participants in the program (N=25).

Characteristics		Participants, n (%)
**Gender**		
	Men	12 (48)
	Women	13 (52)
**Country**		
	Ethiopia	13 (52)
	Rwanda	12 (48)
**Age (years)**		
	<30	7 (28)
	30-39	15 (60)
	40-49	1 (4)
	50-65	2 (8)
**Occupation**		
	Government staff and public sector	12 (48)
	NGOs^a^	10 (40)
	Private sector	2 (8)
	Other	1 (4)
**Education level**		
	Master’s level	19 (76)
	Bachelor’s degree	4 (16)
	Others	2 (8)
**Educational background**		
	Public health	8 (32)
	Nutrition	5 (20)
	Project management	3 (12)
	Medical field	3 (12)
	Agriculture	2 (8)
	Others	4 (16)

^a^NGO: nongovernmental organization.

### Participants’ Knowledge Before and After the Program

On the basis of the results from the preassessment survey, the topic that the participants self-reported having the least previous knowledge about was digital health literacy, and 17 of the 25 (68%, 95% CI 50%-86%) participants expressed having *none* or *very little* knowledge before the program started. In addition, 12 (48%, 95% CI 28%-68%) participants reported having *none* or *very little* knowledge about digital learning before the program started. Corresponding numbers for knowledge level rated as *none* or *very little* for child nutrition and SDGs were 2 (8%, 95% CI 0%-19%) participants and 1 (4%, 95% CI 0%-12%) participant, respectively.

When asked about to what extent they possessed sufficient knowledge and skills to drive change in their organizations before the program started, only 4 (16%, 95% CI 2%-30%) participants replied *to a large extent* or *to a very large extent* for digital health literacy and 5 (20%, 95% CI 4%-36%) participants regarding digital learning. The corresponding numbers for SDGs and child nutrition were 10 (40%, 95% CI 21%-59%) and 13 (52%, 95% CI 32%-73%) participants, respectively (Figure 4).

Least previous knowledge was noted for digital health literacy and digital learning before the program started, but after the program, of the 25 participants, 24 (96%, 95% CI 88%-100%) reported that their knowledge regarding the digital health literacy had increased *to a large extent* or *to a very large extent* after the program, and 23 (92%, 95% CI 81%-100%) reported that their knowledge regarding digital learning increased *to large extent* or *to a very large extent*. Moreover, despite having extensive self-reported knowledge about the SDGs before the program, the postassessment survey showed that all 25 participants reported that their knowledge regarding the SDGs had increased *to a large extent* or *to a very large extent* after the program, whereas 20 (80%, 95% CI 64%-96%) participants reported that their knowledge regarding child nutrition increased *to large extent* or *to a very large extent.* Regarding the participants’ capacity to drive change in their organizations after completing the program, 20 (80%, 95% CI 64%-96%) participants reported that their capacity related to the SDGs had increased *to large extent* or *to a very large extent*, and 20 (80%, 95% CI 64%-96%) reported that their capacity to drive change related to child nutrition increased *to large extent* or *to a very large extent.* Furthermore, 18 (72%, 95% CI 54%-90%) participants reported that their capacity to drive change related to digital health literacy had increased *to a large extent* or *to a very large extent,* and 17 (68%, 95% CI 50%-86%) reported that their capacity to drive change regarding digital learning had increased “to a large extent or to a very large extent” (Figure 5).

### Participants’ Feedback About the Program

Different types of digital learning materials were provided and participants were asked in the postassessment survey about which materials had been the most helpful. Multiple answers were allowed, and almost all participants reported that they found the formative questions helpful (24/25, 96%), followed by module test questions (22/25, 88%), reading the program text and web pages (19/25, 76%), watching videos (18/25, 72%), and web-based workshops (16/25, 64%), and more than half (14/25, 56%) of the participants reported that group assignments were helpful. Overall, approximately three-fourths (19/25, 76%) of the participants felt that their participation in the program was worth the time and effort they had put in.

The postassessment survey included 2 open-ended questions: (1) “Did you miss something in the program?” and (2) “Did the program meet your expectations?” For the first question, more than half (15/25, 60%) of the participants reported that they did not feel anything was missing in the program. The second most common response was that the participants felt that they would have benefited from physical interactions. The second open-ended question yielded more uniform results, in which all but 1 (4%) respondent felt that the program met their expectations. For example, one of the participants wrote the following:

...The content and topics were beyond my expectations. For some modules, it was my first time to hear about them especially in Unit 1 (digital learning) and unit 2 (module 3 and 4) digital health literacy in digital ecosystem was an eye opener. I am looking forward to explore it in my routine work…I am now confidently organizing a Facebook campaign on Covid-19 in Haiti...I do not feel intimidated anymore. Even if it’s something I do not understand, I search online, YouTube and get hands-on...”

Regarding web-based networking and interaction, more than half (14/25, 56%) of the participants reported having been in contact with other program participants on a monthly or weekly basis. In addition, more than half (14/25, 56%) of the participants reported having been in contact on a monthly or weekly basis with teachers participating in the program. No participant had been completely out of touch with the other participants, whereas 1 (4%) participant reported that they had never been in contact with teachers outside of the class.

## Discussion

### Principal Findings

The OneLearns program showed that a digital program is a useful approach to enhance knowledge about issues related to sustainable development among government officials and leaders of NGOs in Ethiopia and Rwanda. All participants (25/25, 100%) reported that their knowledge regarding the SDGs had increased to a large extent or more after the program, and 80% (20/25) reported that their capacity to drive change in their organizations related to the SDGs had increased to a large extent or more. In this program, midlevel to high-ranking government officials and leaders of NGOs were targeted because they influence policy making in their respective countries and often collaborate to bring about changes in communities and policies [30]. Hence, both sectors were included to ensure maximum outreach.

Knowledge, skills, and abilities after completion of a web-based learning experience are common ways to define the effectiveness of a web-based education program [31]; the results from OneLearns show the potential of web-based learning in general and question-based methodology in particular, in Rwanda and Ethiopia. This outcome is in line with previous studies highlighting the benefits of international digital learning to promote good health and well-being as well as advanced education [8,32] and achieving SDGs [33].

Participants also reported that they gained substantial knowledge on topics in which they had initially believed they were already well-versed, such as in the case of the SDGs. At the same time, for topics that the participants reported as having low previous knowledge, such as digital learning and digital health literacy, 92% (23/25) and 96% (24/25), respectively, of the participants reported that their knowledge regarding these issues increased to a large extent or to a very large extent. This indicated the importance of offering advanced professional development to experts in the ministries and NGOs in these countries.

The participants were expected to spend 40 hours on the program over a 3-month period at the same time as they were working in their regular jobs. An advantage of self-study of digital material is that it was possible for the participants to adjust their reading to their own schedules. However, the disadvantage was time management issues, such as interference of daily life routines in studies and some personal problems, which have previously been reported from similar programs [34] and were echoed in the OneLearns program. Moreover, a limitation when conducting web-based learning was poor internet access and connectivity, a well-known challenge in Rwanda and Ethiopia [5] and a frequently reported reason for missed deadlines and poor engagement. For this reason, we offered a physical conference room in Kigali for the final workshop (the bans on meetings in Ethiopia owing to the pandemic prevented the same setup in Addis Ababa) to assure a stable internet connection, but all participants, regardless of physical location, were asked to complete the same tasks during the workshop. Another challenge with professional education in this target group was that the participants had full-time jobs that may have required them to “go out in the field” (as they expressed it) with short notice, which affected their possibilities to partake in program events. We tried to accommodate this by providing extended deadlines, but in the end, it was up to the participants and their employer to prioritize between the program and their ordinary work tasks. This is in line with the study by Heller et al [35], who also noted that those who required more time or had difficulty completing their tasks often reported conflicts with their work schedules.

An example of a digital barrier was that we provided unlimited data packages for all participants for the final workshop, but the poor infrastructure from the local service provider impeded a few participants from having reliable connectivity during this full-day collaborative event. In addition, although weekly reminders were sent containing deadline dates and a general group progress report, deadlines for all 4 module tests had to be extended for some of the participants based on their personal needs. This was done to accommodate the needs of the participants, as the program was a professional development program and not a formal academic course. The foremost goal of the program was to ensure that the participants learned the course material.

An incentive to fulfill all the requirements, announced at the first kickoff seminar, was that the participants in OneLearns were promised a printed and digital certificate at the end of the program. Moreover, a few ambitious and high-performing participants were appointed as course ambassadors. This initiative was implemented after the program started to recognize their efforts and form long-lasting relations between participants and teachers. For example, 2 ambassadors were invited to speak at the kickoff of the second edition of the OneLearns program, which started in September 2021. In addition, all participants were invited to become alumni members of the international network run by the Swedish Institute.

Regarding communication with the participants, the WhatsApp group proved useful and easy to use. All participants had previous experience with the app, and it was their preferred method of communication with the teachers as compared with email. Sending reminders via WhatsApp ensured faster responses or follow-ups than expected from the participants; thus, we increased the use of the app for smoother and faster communication during the course of the program. WhatsApp was used for informal communication among the participants, contributing to networking among professionals in different areas and countries and was available for future communication after the end of the program.

In the open learning initiative platform, data were collected when students answered questions, that is, click-data, and these are used in a machine learning model to predict students’ mastery of the learning objectives [21,22]. Further, these mastery prediction data were used when the teachers prepared the video seminars at the end of each module to assist the teachers; therefore, the focus was on the learning objectives that most students had difficulty understanding. Moreover, 96% (24/25) of the participants indicated that formative questions were helpful in the learning process, and the corresponding number of module test questions was 88% (22/25). This result highlighted the pedagogical method of question-based learning as a successful approach in this context. The click-data were also used after the program to identify which parts of the program needed improvement when preparing for the second edition of OneLearns. In other words, data-driven methodology can be used to improve learning, learning materials, and teaching. For example, for multiple-choice questions, answering alternatives that were not chosen at all or had been answered correctly 100% of the time were interpreted as being too easy or not addressing any sort of misconception, thus not allowing the participants to learn anything new and were therefore changed and improved in the second edition of OneLearns.

### Comparison With Prior Work

Open learning initiative courses using question-based learning methodology are being used at more than a hundred colleges and universities in the United States, but their spread outside North America is limited [36]. We know from previous research that this question-based learning methodology can be very efficient for learners, with the possibility of reducing learning time by 50% for a well-iterated course [22] and 25% for a newly developed course [21], as in our case. Thus, the methodology offered a time-saving alternative for the professional development of experts working full-time in countries with limited resources, as in Rwanda and Ethiopia. However, this depends on internet access, which requires infrastructure. We hope that by letting government officials experience first-hand what state-of-the-art digital learning can offer, they in turn will influence the progression of internet infrastructure in a wise way in their countries.

### Limitations

A potential limitation of this program was selection bias in the recruitment process because participants need to be able to communicate in English, although this is not the official language in Ethiopia and has just recently become one in Rwanda [37]. Although English is widely used in academic and professional settings in both countries, potential key players in decision-making and policy making within the government and NGOs could potentially have been overlooked. Another potential limitation is that we did not assess the participants’ knowledge in the form of formal tests at the beginning and end and therefore, were not able to assess changes in knowledge over time in test results. Now, we rely on their self-reported assessment of their knowledge; thus, future programs should consider formal testing. Another way to assess the capacity for driving change in future programs is to plan for follow-up questionnaires or interviews after the program ended to document the initiatives that the participants had started. Moreover, there was a range of measures of digital health literacy to be used at the research, clinical, organizational, and societal level [38-41] that were not covered in this program. These tools include questions about skills for searching, finding, evaluating, determining relevance, and applying electronic health information to health problems, as well as about engaging with and navigating the health care system. In the next generation of OneLearns, the participants will learn these methods and how to use them. In addition, future pedagogical research may include experiments on the effect of sending reminders and program process reports.

The next step for participants is to implement what they learned and build capacity for change in their organizations. If done on a large scale, it will take time, but may include, for example, increased use of digital learning in schools, universities, and other educational organizations. Digital health literacy may lead to increased ability of the general population and professionals to seek information on self-care, access to care, increased use of internet-based medical consultations to reduce emergency department visits, and electronic receipts of drugs [37]. In addition, a broad knowledge of child nutrition will allow for future programs with intersectoral collaborations to address the multifactorial nature of malnutrition. Finally, developing the infrastructure for digitalization is of high priority for both Rwanda and Ethiopia, but it is costly and requires trained human resources. Rwanda has a national information and communication infrastructure development plan aimed at developing and facilitating the establishment and growth of its ICT sector [42], and Ethiopia has a national ICT sector policy and strategy [43].

### Conclusions

Digital learning based on question-based learning methodology is a useful method to increase knowledge and the capacity to drive change related to sustainable development among government officials and decision-makers at NGOs in Rwanda and Ethiopia.

## Appendix

Multimedia Appendix 1 A layout of web-based program.

Multimedia Appendix 2 Sticky note function in the Miro program.

Multimedia Appendix 3 The preassessment survey questionnaire.

Multimedia Appendix 4 The postassessment survey questionnaire.

## Abbreviations

ICT: information communication technology
NGO: nongovernmental organization
OneLearns: Online Education for Leaders in Nutrition and Sustainability
SDG: sustainable development goal
