# Supplementary material for: A Web-Based Program About Sustainable Development Goals Focusing on Digital Learning, Digital Health Literacy, and Nutrition for Professional Development in Ethiopia and Rwanda: Development of a Pedagogical Method
Source: JMIR Form Res. 2022 Dec 5;6(12):e36585. doi: 10.2196/36585 (PMC9764148; doi:10.2196/36585)
Supplement: Multimedia Appendix 1 [file formative_v6i12e36585_app1.pdf]

A layout of modules, learning objectives, chapters, and sections per unit in the program as presented to the participants.

Modules[a]	Learning objectives	Chapters	Sections
**Unit 1: Effective digital learning**			
A. Learning online	Apply skills for self-directed learning	Becoming a web-based learner	• Skills for self-directed learning<br>• Plan-Monitor-Evaluate<br>• Critical questioning to support your learning
B. Effective digital learning	Comprehend how learning occurs	Learning	• Knowledge<br>• Learning theories<br>• Steps to learning<br>• Learning levels<br>• The Dunning-Kruger effect
	Comprehend how efficient online learning is constructed	Effective learning	• Online learning<br>• Active learning<br>• Skill-based feedback<br>• Learning curves
**Unit 2: Digital health literacy**			
C. Introduction to digital health literacy	Describe health literacy and related issues	Health literacy and related issues	• Definition and conceptualization of health literacy<br>• Determinants of health literacy<br>• How health literacy links to health outcomes<br>• The essential skills of health literacy
	Recognize the transformation of	Health literacy in the digital era	• Components of digital health literacy

		health literacy to digital health literacy		• Barriers to achieving digital health literacy<br>• The strategies to address challenges of digital health literacy<br>• Information privacy in eHealth context
		Describe ways of working on the web in a secure manner	Internet security	• Introduction to internet security<br>• Computer security: passwords<br>• Computer security: phishing<br>• Dealing with malware
		Recognize computer literacy	Computer literacy basics	• Basic computer & internet communication fundamentals<br>• Recognizing health care–web-based work environment
	D. Fundamentals of digital literacy in contemporary health care	Recognize fundamental concepts associated with change in health care digitization	Digital health fundamentals	• Digital health user engagement<br>• Appraising digital health & change management<br>• Digital health relevance
**Unit 3: Nutrition and the sustainable development goals**				
	E. Nutrition and Sustainability	Recognize the functionality of the sustainable development goals in public health and sustainable development;	Understanding the sustainable development goals	• Introduction to sustainable development and agenda 2030<br>• Evaluating the sustainable development goals<br>• Key concepts in public health nutrition: transition models and disease burden

		Recognize the environmental and societal contributors to sustainable nutrition; discuss some possible innovative solutions and rethinking strategies that can address nutrition and sustainability;	Components of sustainable nutrition	• Nutrition, society, and the environment<br>• Nutrition and water<br>• Nutrition and gender equity<br>• Rethinking nutrition
		Describe stunting and its relation to physical and cognitive development; Describe young children's nutritional needs, with a focus on the first 1000 days of life; choose healthy food items that provide sufficient energy and nutrients to cover children's needs;	Child nutrition	• Nutritional needs of young children<br>• Stunting

[a] Empty cell indicates that there are several learning outcomes per modules.
