# Supplementary material for: A Web-Based Program About Sustainable Development Goals Focusing on Digital Learning, Digital Health Literacy, and Nutrition for Professional Development in Ethiopia and Rwanda: Development of a Pedagogical Method
Source: JMIR Form Res. 2022 Dec 5;6(12):e36585. doi: 10.2196/36585 (PMC9764148; doi:10.2196/36585)

The use of the Sticky Note function in the Miro program during the final workshop. This was an interactive brainstorming session among all the participants about barriers, facilitators and solutions linked to different SDGs and a way for the participants to put their new knowledge in use.

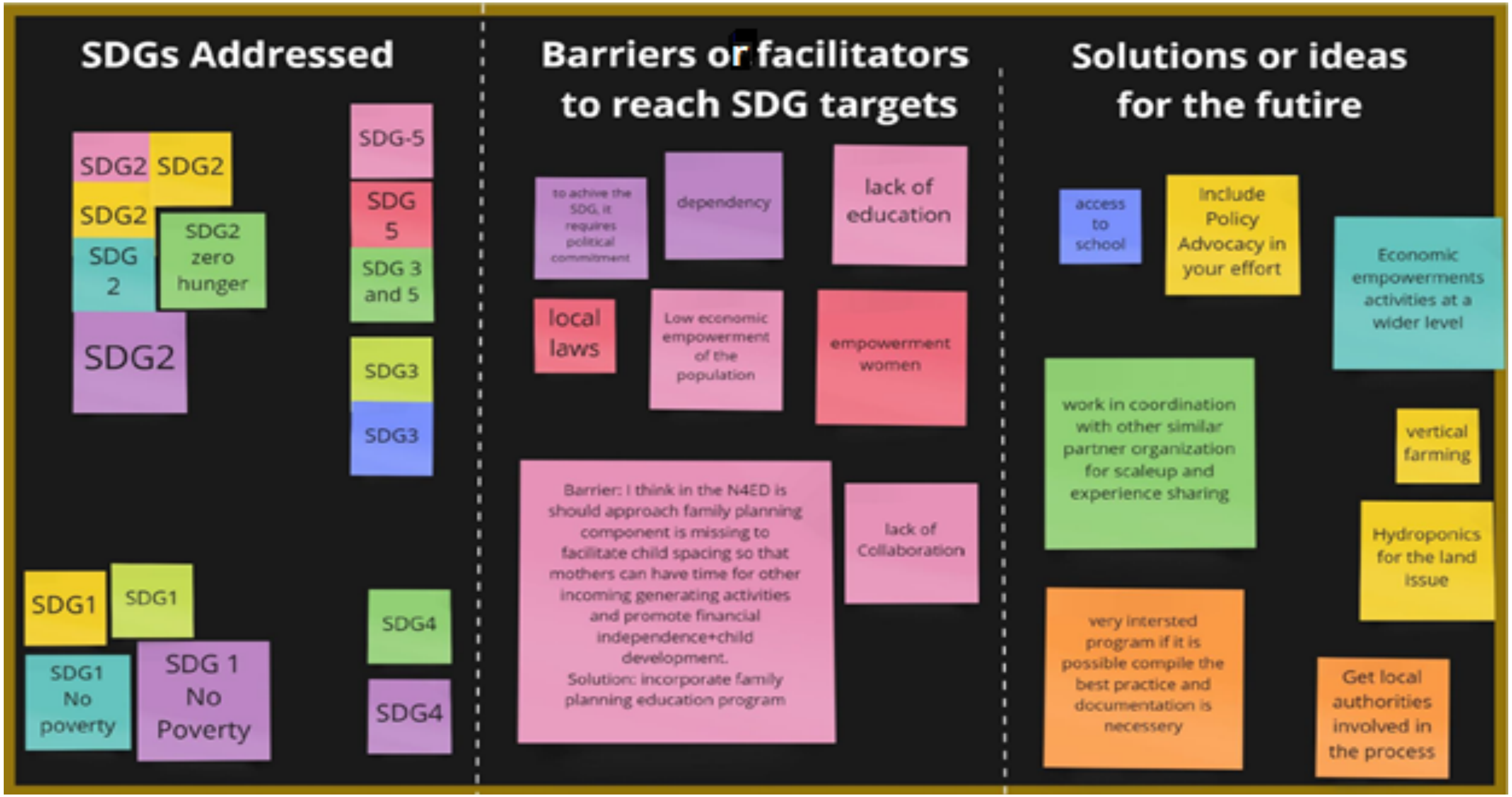

Supplement: Multimedia Appendix 2 [file formative_v6i12e36585_app2.pdf]
