# Supplementary material for: A Web-Based Program About Sustainable Development Goals Focusing on Digital Learning, Digital Health Literacy, and Nutrition for Professional Development in Ethiopia and Rwanda: Development of a Pedagogical Method
Source: JMIR Form Res. 2022 Dec 5;6(12):e36585. doi: 10.2196/36585 (PMC9764148; doi:10.2196/36585)
Supplement: Multimedia Appendix 3 [file formative_v6i12e36585_app3.pdf]

Web-based pre-assessment questionnaire for OneLearns

Welcome to the course One Learns about digital learning, digital health literacy, sustainable development goals, and child nutrition. Please help us improve the course by filling out this short questionnaire. A similar questionnaire will be sent out after the course. Thank you and we are looking forward to seeing your soon!

The One Learns team at the Royal Institute of Technology and Mälardalen University, Sweden

Instructions

The questionnaire includes 14 questions and will only take a few minutes to answer. Please select the answer alternative that you feel is the most appropriate for your situation. N/A means "Not Applicable" and can be used if you feel that none of the alternatives apply to you. We will only store the answers from the questionnaire until the end of this year so that we will have time to revise and improve the course. Then we will delete the data. The overall result from the questionnaires will be summarized on a group level, and individual answers will not be possible to trace in the final material.

Please, indicate your age:

- Below 30 ☐
- 30-39 ☐
- 40-49 ☐
- 50-65 ☐
- Above 65 ☐
- Don't know/ Don´t want to answer ☐

What is your sex?

- Female ☐
- Male ☐
- Don't know/ Don´t want to answer ☐

In which country do you work?

Ethiopia ☐

Rwanda ☐

N/A ☐

Which sector are you currently working in?

Public sector ☐

Private sector ☐

NGO ☐

Civil Society ☐

Unemployed ☐

Other ☐

N/A ☐

Please rate your level of knowledge about the following issues:

	**None**	**Very little knowledge**	**Some knowledge**	**Good knowledge**	**Very good knowledge**	**N/A**
Digital learning	☐	☐	☐	☐	☐	☐
Digital health literacy	☐	☐	☐	☐	☐	☐
Child nutrition	☐	☐	☐	☐	☐	☐
Sustainable Development Goals	☐	☐	☐	☐	☐	☐

To what extent do you have sufficient knowledge and skills to drive change regarding:

	Not at all	To a small extent	To some extent	To a large extent	To a very large extent	N/A
Digital learning	☐	☐	☐	☐	☐	☐
Digital health literacy	☐	☐	☐	☐	☐	☐
Child nutrition	☐	☐	☐	☐	☐	☐
Sustainable Development Goals	☐	☐	☐	☐	☐	☐

To what extent do you have the confidence to drive change regarding:

	Not at all	To a small extent	To some extent	To a large extent	To a very large extent	N/A
Digital learning	☐	☐	☐	☐	☐	☐
Digital health literacy	☐	☐	☐	☐	☐	☐
Child nutrition	☐	☐	☐	☐	☐	☐
Sustainable Development Goals	☐	☐	☐	☐	☐	☐

To what extent do you, in your daily work, work with:

	Not at all	To a small extent	To some extent	To a large extent	To a very large extent	N/A
Digital learning	☐	☐	☐	☐	☐	☐
Digital health literacy	☐	☐	☐	☐	☐	☐
Child nutrition	☐	☐	☐	☐	☐	☐
Sustainable Development Goals	☐	☐	☐	☐	☐	☐

To what extent does consideration of the following issues affect decisions you make in your daily work?

	Not at all	To a small extent	To some extent	To a large extent	To a very large extent	N/A
Digital learning	☐	☐	☐	☐	☐	☐
Digital health literacy	☐	☐	☐	☐	☐	☐
Child nutrition	☐	☐	☐	☐	☐	☐
Sustainable Development Goals	☐	☐	☐	☐	☐	☐

To what extent are the following issues prioritised in your company/authority/organisation:

	Not at all	To a small extent	To some extent	To a large extent	To a very large extent	N/A
Digital learning	☐	☐	☐	☐	☐	☐
Digital health literacy	☐	☐	☐	☐	☐	☐
Child nutrition	☐	☐	☐	☐	☐	☐
Sustainable Development Goals	☐	☐	☐	☐	☐	☐

To what extent are the following issues prioritized at your local work place, for example your unit/department:

	Not at all	To a small extent	To some extent	To a large extent	To a very large extent	N/A
Digital learning	☐	☐	☐	☐	☐	☐
Digital health literacy	☐	☐	☐	☐	☐	☐
Child nutrition	☐	☐	☐	☐	☐	☐
Sustainable Development Goals	☐	☐	☐	☐	☐	☐

How often do you consult or interact with networks relating to:

	Never	Very rarely	Occasionally (a few times per year)	Regularly (at least monthly)	Frequently (at least weekly)	N/A
Digital learning	☐	☐	☐	☐	☐	☐
Digital health literacy	☐	☐	☐	☐	☐	☐
Child nutrition	☐	☐	☐	☐	☐	☐
Sustainable Development Goals	☐	☐	☐	☐	☐	☐

To what extent are you committed to increase awareness and bring change to your organization regarding:

	Not at all	To a small extent	To some extent	To a large extent	To a very large extent	N/A
Digital learning	☐	☐	☐	☐	☐	☐
Digital health literacy	☐	☐	☐	☐	☐	☐
Child nutrition	☐	☐	☐	☐	☐	☐
Sustainable Development Goals	☐	☐	☐	☐	☐	☐

Examples are very welcome, please comment in the text box below

....

....

....

What are your expectations on this course?

....

....

....

Thank you for taking the time to answer these questions! Your answers have been saved and you can close this window on your computer. The One Learns team at the Royal Institute of Technology and Mälardalen University
