# Supplementary material for: A Web-Based Program About Sustainable Development Goals Focusing on Digital Learning, Digital Health Literacy, and Nutrition for Professional Development in Ethiopia and Rwanda: Development of a Pedagogical Method
Source: JMIR Form Res. 2022 Dec 5;6(12):e36585. doi: 10.2196/36585 (PMC9764148; doi:10.2196/36585)
Supplement: Multimedia Appendix 4 [file formative_v6i12e36585_app4.pdf]

Web-based post-assessment questionnaire for OneLearns

Thank you for participating in the course One Learns about digital learning, digital health literacy, sustainable development goals, and child nutrition. Please help us improve the course by filling out this short questionnaire. Thank you!

The One Learns team at the Royal Institute of Technology and Mälardalen University, Sweden.

Instructions

The questionnaire includes 10 questions and will only take a few minutes to answer. Please select the answer alternative that you feel is the most appropriate for your situation. N/A means "Not Applicable" and can be used if you feel that none of the alternatives apply to you. We will only store the answers from the questionnaire until the end of this year so that we will have time to revise and improve the course. Then we will delete the data. The overall result from the questionnaires will be summarized on a group level, and individual answers will not be possible to trace in the final material.

To what extent did your knowledge about the following issues increase as a result of your participation in the programme regarding:

	**Not at all**	**To a small extent**	**To some extent**	**To a large extent**	**To a very large extent**	**Already expert before the course**	**N/A**
Digital learning	☐	☐	☐	☐	☐	☐	☐
Digital health literacy	☐	☐	☐	☐	☐	☐	☐
Child nutrition	☐	☐	☐	☐	☐	☐	☐
Sustainable Development Goals	☐	☐	☐	☐	☐	☐	☐

To what extent did participating in the programme increase your capacity to drive change in your organization regarding:

	Not at all	To a small extent	To some extent	To a large extent	To a very large extent	N/A
Digital learning	☐	☐	☐	☐	☐	☐
Digital health literacy	☐	☐	☐	☐	☐	☐
Child nutrition	☐	☐	☐	☐	☐	☐
Sustainable Development Goals	☐	☐	☐	☐	☐	☐

Looking back, was your participation in the training programme worth the time, money and effort you and your organisation spent on it?

Not at all	☐
To some extent	☐
Almost but not quite	☐
It was worth it all	☐
It was worth much more	☐
N/A	☐

How often have you been in contact with:

	Never	Very rarely	Occasionally (a few times)	Regularly (at least monthly)	Frequently (at least weekly)	N/A
The other participants in the programme	☐	☐	☐	☐	☐	☐
The teachers/instructors at the programme	☐	☐	☐	☐	☐	☐
The Swedish Institute	☐	☐	☐	☐	☐	☐
Other contacts made through the programme	☐	☐	☐	☐	☐	☐

Which parts of the course was most helpful for you:

Reading the course text and webpages	☐
Looking at movies and clips	☐
Formative questions in the learning material	☐
Module test questions	☐
Group assignments	☐
Online workshops	☐
Others	☐

Did you miss something in the course?

....

....

....

Did the course meet your expectations?

....

....

....

Thank you for taking the time to answer these questions! Your answers have been saved and you can close this window on your computer. The One Learns team at the Royal Institute of Technology and Mälardalen University.
